# Hepatocyte-Specific Deficiency of BAP31 Amplified Acetaminophen-Induced Hepatotoxicity via Attenuating Nrf2 Signaling Activation in Mice

**DOI:** 10.3390/ijms221910788

**Published:** 2021-10-05

**Authors:** Jie Zhao, Xiaotong Lv, Yan Huo, Xiaodi Hu, Xiaochen Li, Shizhuo Sun, Xin Zhao, Xuewei Kong, Jialin Xu

**Affiliations:** Institute of Biochemistry and Molecular Biology, College of Life and Health Sciences, Northeastern University, Shenyang 110819, China; zhao_jie95@163.com (J.Z.); 5851249love@sina.cn (X.L.); huoyan_hy@163.com (Y.H.); 18363973230@163.com (X.H.); qylengju@163.com (X.L.); zwmty1@163.com (S.S.); 1901402@stu.neu.edu.cn (X.Z.); 15037601822@163.com (X.K.)

**Keywords:** BAP31, acetaminophen, oxidative stress, Nrf2

## Abstract

Liver-specific deficiency of B-cell receptor-associated protein 31 knockout mice (BAP31-LKO) and the littermates were injected with acetaminophen (APAP), markers of liver injury, and the potential molecular mechanisms were determined. In response to APAP overdose, serum aspartate aminotransferase and alanine aminotransferase levels were increased in BAP31-LKO mice than in wild-type controls, accompanied by enhanced liver necrosis. APAP-induced apoptosis and mortality were increased. Hepatic glutathione was decreased (1.60 ± 0.31 μmol/g tissue in WT mice vs. 0.85 ± 0.14 μmol/g tissue in BAP31-LKO mice at 6 h, *p* < 0.05), along with reduced glutathione reductase activity and superoxide dismutase; while malondialdehyde was significantly induced (0.41 ± 0.03 nmol/mg tissue in WT mice vs. 0.50 ± 0.05 nmol/mg tissue in BAP31-LKO mice for 6 h, *p* < 0.05). JNK signaling activation and APAP-induced hepatic inflammation were increased in BAP31-LKO mice. The mechanism research revealed that BAP31-deficiency decreased *Nrf2* mRNA stability (half-life of *Nrf2* mRNA decreased from ~1.3 h to ~40 min) and *miR-223* expression, led to reduced nuclear factor erythroid 2-related factor 2 (Nrf2) signaling activation and antioxidant genes induction. BAP31-deficiency decreased mitochondrial membrane potentials, reduced mitochondria-related genes expression, and resulted in mitochondrial dysfunction in the liver. Conclusions: BAP31-deficiency reduced the antioxidant response and Nrf2 signaling activation via reducing *Nrf2* mRNA stabilization, enhanced JNK signaling activation, hepatic inflammation, and apoptosis, amplified APAP-induced hepatotoxicity in mice.

## 1. Introduction

Acetaminophen (APAP) is an over-the-counter antipyretic and analgesic drug with an excellent safety profile when administered properly. However, APAP overdose causes severe and acute liver injury [1]. In the United States, there are approximately 6000 acute liver injuries caused by APAP overdose every year, with a high mortality rate of up to 8%, which seriously affects human health [2]. Mechanistically, a proper amount of APAP is metabolized by cytochrome P450 enzymes into the reactive metabolite of N-acetyl-p-benzoquinonimine (NAPQI), which is then detoxified by conjugation with hepatic glutathione (GSH) [3]. Therefore, excessive APAP can deplete liver GSH in the plasma, leading to the covalent binding of NAPQI to mitochondrial proteins, which in turn triggers the initiation of mitochondrial dysfunction, excessive reactive oxygen species (ROS) production, c-Jun N-terminal kinase (JNK) signaling activation and DNA damage, results in liver cellular destruction, apoptosis and necrosis ultimately [4]. Despite the intensive efforts that have been expended in the past decades, the pathophysiological process of APAP overdose still remains poorly understood. The therapeutic options for treating APAP-induced hepatotoxicity are rather limited.

B-cell receptor-associated protein 31 (BAP31) is a ubiquitously expressed and integral protein in the endoplasmic reticulum (ER) membrane. Previous studies reported that BAP31 promoted vesicle trafficking of proteins of class I molecules [5] and tetraspanins [6], suggesting the function of BAP31 involved in immunity and apoptosis. BAP31 depletion reduced T cell activation via regulating T-cell antigen receptor (TCR) signaling in mice [7]. BAP31 expression was up-regulated in stages I, II, and III of cervical cancer patients. Depletion of BAP31 inhibited cervical cancer cell invasion, and migration prevented cervical cancer progression and metastasis [8]. Our recent publications reported that liver-specific deficiency of BAP31 induced Sterol regulatory element binding protein 1 (SREBP1) signaling activation, promoted liver steatosis and worsened insulin resistance in high-fat diet (HFD)-induced obese mice; and increased tunicamycin-induced ER stress reduced the rate of fatty acid oxidation, which promoted tunicamycin-induced liver steatosis, along with increased liver injury markers of serum alanine transaminase (ALT) and aspartate transaminase (AST) in both animal models [9,10]. In addition, the patients with *BAP31* and *ATP binding cassette subfamily D member 1* (*ABCD1*) deletion exhibited increased serum ALT and AST levels than healthy controls [11], suggesting the important roles of BAP31 in maintaining physiological function for the liver organ. However, relatively fewer academic researches of BAP31 focused on the protective roles against APAP-induced liver injury are available, and the molecular mechanism is still needed to be completed then.

Nuclear factor erythroid 2-related factor 2 (Nrf2) is an essential transcription factor that mediates cellular anti-oxidative response by modulating an array of detoxifying and antioxidant defense genes in the liver. Numerous evidence indicated that Nrf2 signaling is involved in the pathogenesis of various liver diseases, including APAP-induced liver injury [12,13]. To explore the protective roles of BAP31 against liver injury, and to investigate the molecular mechanisms under BAP31 modulation, male liver-specific deficiency of BAP31 conditional knockout mice (BAP31-LKO), and wild-type littermates (WT) were injected with APAP, markers of liver injury were determined. The antioxidant system and Nrf2 signaling activation were also evaluated. The current study demonstrated that BAP31-deficiency reduced *Nrf2* mRNA stability, inhibited Nrf2 signaling activation and the antioxidant response, increased hepatic inflammation and oxidative stress, promoted the mice more susceptible to APAP-induced hepatotoxicity.

## 2. Results

### 2.1. BAP31-Deficiency Increased APAP-Induced Liver Injury

**Figure 1:**
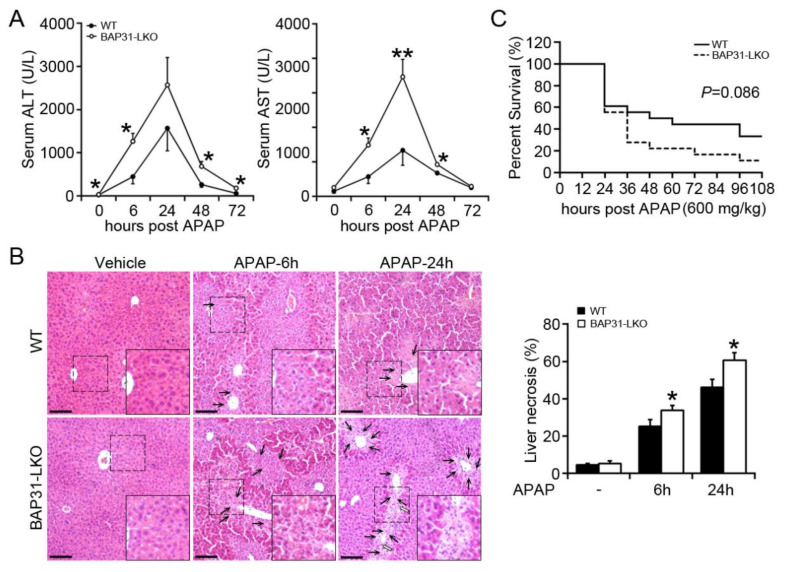
BAP31-deficiency increased APAP-induced liver injury. (**A**) 10-week-old male BAP31-LKO mice and the littermates were i.p. injected with 300 mg/kg APAP after overnight food deprivation. Serum ALT and AST levels were measured at various time points. *n* = 5–7 per group. (**B**) Representative images of H/E staining of liver tissues (left panel). Area of necrosis (% of area) was determined (right panel). Black arrows indicate necrotic hepatocytes. White arrows indicate neutrophil infiltration. Scar bar = 100 μm. *n* = 4 per group. (**C**) The survival rate of WT and BAP31-LKO mice at different time points after injection with a lethal dose of APAP (600 mg/kg). *n* = 18 per group. Data are expressed as mean ± SE. *, *p* < 0.05, **, *p* < 0.01, BAP31-LKO compared to WT mice.

To investigate the roles of BAP31 in APAP-induced acute liver injury, we performed serological and histological analysis between BAP31-LKO mice and WT littermates treated with a single dose of APAP (300 mg/kg). As expected, serum ALT and AST levels were elevated progressively and reached to peak at 24 h after APAP administration. Notably, the amplitude of APAP-induced elevation of these two markers in BAP31-LKO mice was substantially higher than in WT littermates (Figure 1A). Liver injury was characterized by necrosis in the centrilobular region, a hallmark of APAP toxicity. Histological analysis revealed that BAP31-LKO mice had higher hepatic necrosis and extensive multifocal neutrophil infiltration (Figure 1B). In addition, mice were challenged with a lethal dose of APAP, and the mortality rate was determined. As shown in Figure 1C, BAP31-LKO mice exhibited a lower survival rate than WT littermates (11.1% vs. 33.3%, *p* = 0.086), indicating a protective role of BAP31 involved in APAP-induced liver injury and mortality (Figure 1C).

### 2.2. BAP31-Deficiency Amplified APAP-Induced GSH Depletion and Hepatotoxicity

**Figure 2:**
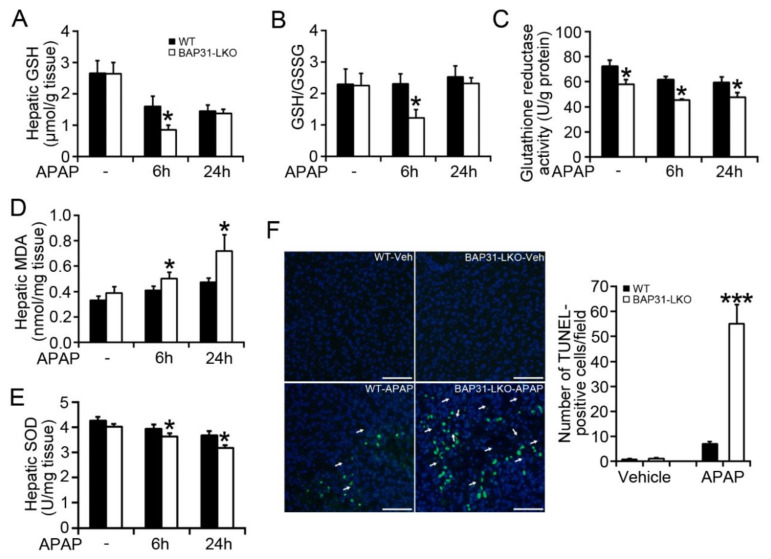
BAP31-deficiency amplified APAP-induced GSH depletion and hepatotoxicity. (**A**) Hepatic GSH, (**B**) GSH/GSSG ratio, (**C**) glutathione reductase activity, (**D**) hepatic MDA, and (**E**) hepatic SOD were determined in each group of mice. *n* = 5–7 per group. (**F**) Representative images of TUNEL staining in each group of mice 24 h post APAP injection (left panel). Arrows indicate the apoptotic hepatocytes (green staining). Nuclei were counterstained with DAPI (blue). Semi-quantification of the number of TUNEL-positive cells per field (right panel). Scar bar = 100 μm. *n* = 4 per group. Data are expressed as mean ± SE. *, *p* < 0.05, ***, *p* < 0.001, BAP31-LKO compared to WT mice.

There is a significant reduction in hepatic GSH in BAP31-LKO mice (0.85 ± 0.14 μmol/g tissue) when compared to WT controls (1.60 ± 0.31 μmol/g tissue) 6 h post APAP administration (*p* < 0.05, Figure 2A), along with a similar reduction in GSH/GSSG ratio (Figure 2B), due to the reduced glutathione reductase activity in the liver (Figure 2C). Hepatic malondialdehyde (MDA) levels were significantly increased in BAP31-LKO mice (0.50 ± 0.05 nmol/mg tissue vs. 0.41 ± 0.03 nmol/mg tissue in WT mice for 6 h, *p* < 0.05; and 0.72 ± 0.13 nmol/mg tissue vs. 0.47 ± 0.03 nmol/mg tissue in WT mice for 24 h, *p* < 0.05. Figure 2D). The antioxidant enzyme of superoxide dismutase (SOD) was decreased in WT mice by APAP treatment and reduced even more in BAP31-LKO mice (Figure 2E), suggesting that BAP31-deficiency worsened GSH depletion and APAP-induced oxidative stress. DNA fragmentation, a characteristic feature of APAP-induced hepatocyte death [14], was further induced in APAP-treated BAP31-LKO mice as compared to WT controls (*p* < 0.001, Figure 2F).

### 2.3. BAP31-Deficiency Reduced Antioxidant and Detoxification Genes Expression

**Figure 3:**
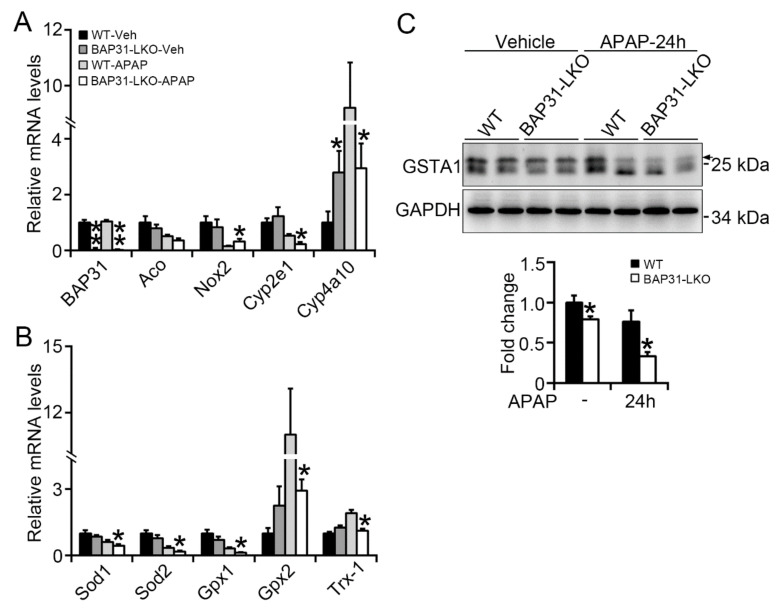
BAP31-deficiency reduced antioxidant and detoxification genes expression. BAP31-LKO mice and the littermates were i.p. injected APAP (300 mg/kg) for 24 h. Liver tissues were harvested, and the total RNA was extracted. The transcriptional levels of (**A**) *BAP31*, *Aco*, *Nox2*, *Cyp2e1*, and *Cyp4a10*, (**B**) *Sod1*, *Sod2*, *Gpx1*, *Gpx2*, and *Trx-1* were determined. The relative mRNA levels were normalized by *18S* rRNA levels. *n* = 5–7 per group. (**C**) The protein levels of GSTA1 were determined using immunoblot analysis in livers from WT and BAP31-LKO mice 24 h post APAP injection. GAPDH was used as the loading control. Data are expressed as mean ± SE. *, *p* < 0.05, **, *p* < 0.01, BAP31-LKO compared to WT mice.

Increased oxidative stress is due to an excessive ROS accumulation, occurring as a result of the imbalance between ROS-generating and -scavenging systems. We determined the expression levels of several key genes, including *acyl-coenzyme A oxidase* (*Aco*), *NADPH oxidase-2* (*Nox2*), and *cytochrome P450 2e1* (*Cyp2e1*), *Cyp4a10*, as well as *Sod1*, *Sod2*, *glutathione peroxidase 1* (*Gpx1*), *Gpx2*, *thioredoxin-1* (*Trx-1*), which have been suggested involved in both processes and modulated by Nrf2 signaling [15,16]. *BAP31* mRNA levels were significantly reduced in BAP31-LKO mice than in WT controls and exhibited no obvious difference after APAP administration. The ROS-generating genes, including *Aco*, *Nox2*, *Cyp2e1*, and *Cyp4a10*, displayed inconsistent expression patterns (no significant difference, increase, decrease, or decrease) in BAP31-LKO mice when compared to WT controls after APAP administration (Figure 3A). However, the antioxidant genes of *Sod1*, *Sod2*, *Gpx-1*, *Gpx2*, and *Trx-1* were significantly reduced (Figure 3B). These results suggested that the increased oxidative stress in BAP31-LKO mice was attributed to the defective ROS scavenging system, not due to the ROS-generating system in the liver. Glutathione S-transferase alpha 1 (GSTA1) is a detoxification enzyme and plays a key role in the protection against oxidative stress, and also was reduced in BAP31-LKO mice, suggesting the important roles of the BAP31 gene in scavenging oxidative stress after APAP administration (Figure 3C).

### 2.4. BAP31-Deficiency Increased APAP-Induced Inflammatory Response

**Figure 4:**
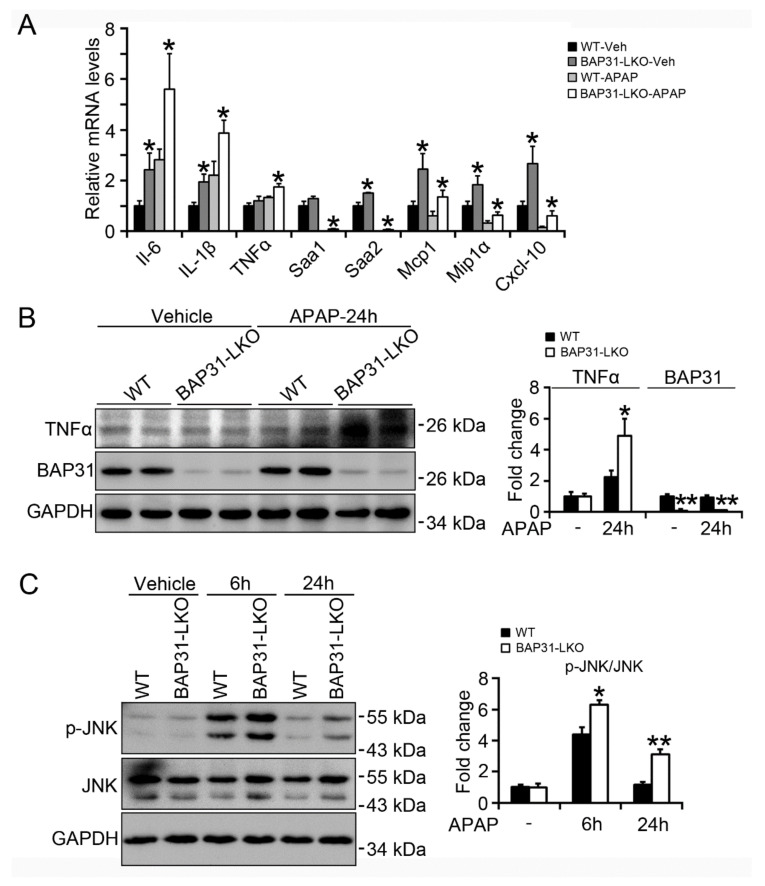
BAP31-deficiency increased APAP-induced inflammatory response. (**A**) The transcriptional levels of *IL-6*, *IL-1β*, *TNFα*, *Saa1*, *Saa2*, *Mcp1*, *Mip1α*, and *Cxcl-10* were determined. The relative mRNA levels were normalized by *18S* mRNA levels. *n* = 5–7 per group. (**B**) The protein levels of TNFα and BAP31 were determined in livers from WT and BAP31-LKO mice 24 h post APAP injection. (**C**) The phosphorylation levels of JNK were measured in livers from WT and BAP31-LKO mice 6 and 24 h post APAP injection. GAPDH and JNK were used as the loading control. Data are expressed as mean ± SE. *, *p* < 0.05, **, *p* < 0.01, BAP31-LKO compared to WT mice.

The mRNA levels of the pro-inflammatory markers, including *interleukin-6* (*IL-6*), *IL-1β*, *tumor necrosis factor α* (*TNFα*), *serum amyloid a 1* (*Saa1*), *Saa2*, *monocyte chemoattractant protein* (*Mcp1*), *macrophage inflammatory protein 1α* (*Mip1α*), and *C-X-C motif chemokine ligand 10* (*Cxcl-10*) were determined by quantitative real-time PCR, which displayed significant induction of inflammatory response genes in BAP31-LKO mice as compared to WT littermates, led to enhanced hepatic inflammation and promoted hepatotoxicity upon APAP overdose (Figure 4A). Consistently, TNFα protein levels were significantly induced in APAP-treated BAP31-LKO mice (Figure 4B). APAP administration induces acute liver injury, activates the JNK signaling pathway, and in turn accelerates hepatocyte apoptosis and the inflammatory response. The phosphorylation levels of JNK (p-JNK) increased significantly in WT controls after APAP administration and increased even higher in BAP31-LKO mice (Figure 4C), suggesting that BAP31 depletion in hepatocytes promoted JNK signaling activation and increased APAP-induced inflammatory response in mice.

### 2.5. BAP31-Deficiency Reduced Nrf2 Signaling Activation

**Figure 5:**
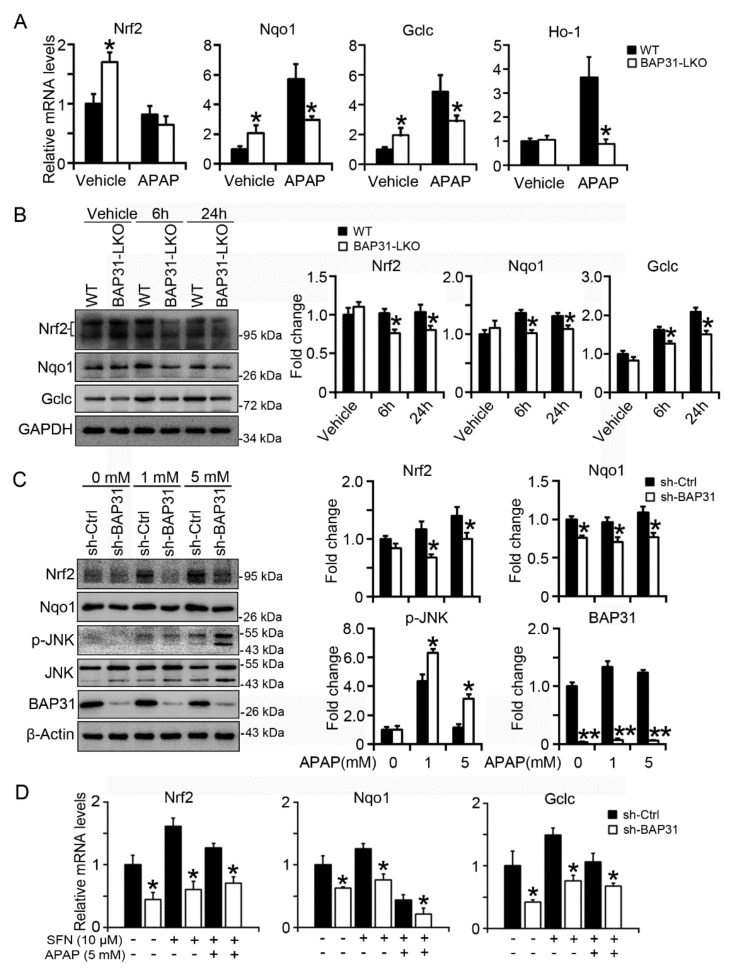
BAP31-deficiency reduced Nrf2 signaling activation. (**A**) The transcriptional levels of *Nrf2*, *Nqo1*, *Gclc*, and *Ho-1* were measured in WT, and BAP31-LKO mice post APAP administration. The relative mRNA levels were normalized by *18S* rRNA levels. *n* = 5–7 per group. (**B**) The protein levels of Nrf2, Gclc, and Nqo1 were determined. GAPDH was used as the loading control. Data are expressed as mean ± SE. *, *p* < 0.05, BAP31-LKO compared to WT mice. (**C**) HepG2 cells with targeted deficiency of BAP31 were treated with APAP (1 and 5 mM) for 24 h, and then the protein levels of Nrf2, Nqo1, p-JNK, and JNK were determined. β-Actin and JNK were used as the loading control. (**D**) BAP31-deficiency reduced sulforaphane induction of *Nrf2*, *Nqo1*, and *Gclc* mRNA levels in HepG2 cells. HepG2 cells were treated with sulforaphane (SFN, 10 μM) and acetaminophen (APAP, 5 mM) for 24 h, and then the mRNA levels of *Nrf2*, *Nqo1*, and *Gclc* were determined using quantitative real-time PCR. The relative mRNA levels were normalized by *18S* rRNA levels. Data are expressed as mean ± SE. *, *p* < 0.05, **, *p* < 0.01, sh-BAP31 compared to sh-Ctrl cells.

The mRNA levels of Nrf2 targets of *NAD(P)H quinone dehydrogenase 1* (*Nqo1*), *glutamate-cysteine ligase, catalytic subunit* (*Gclc*), and *heme oxygenase-1* (*Ho-1*) were significantly reduced in BAP31-LKO mice than in WT littermates after APAP treatment, accompanied by the insignificant reduction in *Nrf2* mRNA levels (Figure 5A). The protein levels of Nrf2, as well as Nqo1 and Gclc, were also significantly reduced, suggesting that Nrf2 signaling was reduced in BAP31-LKO mice livers (Figure 5B). Additionally, these observations were confirmed from *in vitro* studies. HepG2 cells with targeted deficiency of BAP31 were treated with APAP (1 and 5 mM) for 24 h. Compared to the sh-Ctrl group, sh-BAP31 cells exhibited reduced Nrf2 and Nqo1 protein levels and induced p-JNK levels after APAP treatment (Figure 5C). Sulforaphane (SFN), one of the Nrf2 signaling activators, induced *Nrf2*, *Nqo1*, and *Glclc* mRNA levels, but the induction was reduced in sh-BAP31 cells (Figure 5D), demonstrated that BAP31-deficiency reduced Nrf2 signaling activation.

### 2.6. BAP31-Deficiency Reduced mRNA Stability and Impaired Nrf2 Signaling Induction

**Figure 6:**
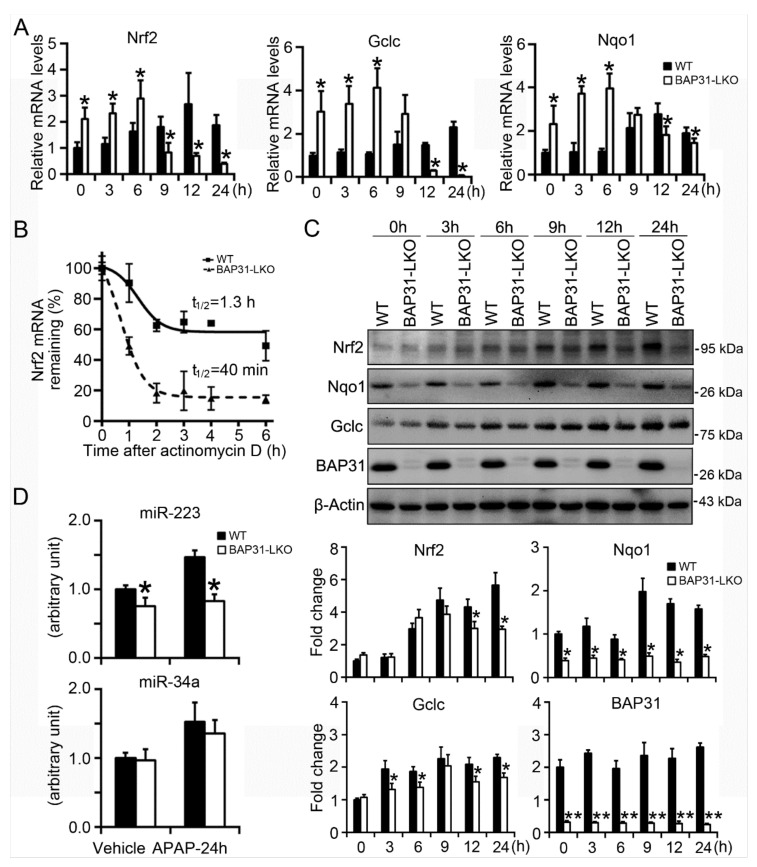
BAP31-deficiency reduced mRNA stability and impaired Nrf2 signaling induction. (**A**) Primary mouse hepatocytes were isolated from WT and BAP31-LKO mice and then were treated with APAP (5 mM) for 0, 3, 6, 9, 12, and 24 h. The mRNA levels of *Nrf2*, *Nqo1*, and *Gclc* were determined. (**B**) BAP31-deficiency reduced *Nrf2* mRNA stability. Actinomycin D was added to the culture medium for the indicated time. *Nrf2* mRNA levels were determined, and the half-life was calculated to assess mRNA decay kinetics. (**C**) Cellular protein was extracted from the primary hepatocytes, and the protein levels of Nrf2, Nqo1, Gclc, and BAP31 were determined. β-Actin was used as the loading control. *, *p* < 0.05, **, *p* < 0.01, BAP31-LKO hepatocytes compared to WT hepatocytes. (**D**) The expression levels of *miR-223* and *miR-34a* were determined in liver tissues. U6 was selected as the internal control. *n* = 5 per group. Data are expressed as mean ± SE. *, *p* < 0.05, BAP31-LKO compared to WT mice.

In order to explore the underlying mechanism of reduced Nrf2 signaling activation in BAP31-LKO mice, the primary hepatocytes were isolated and treated with APAP (5 mM) for different time courses. As shown in Figure 6A, starting from 9 h after APAP treatment, *Nrf2* mRNA was decreased in BAP31-LKO hepatocytes than in WT hepatocytes; *Gclc* and *Nqo1* were reduced in BAP31-LKO hepatocytes than in WT hepatocytes starting from 12 h after APAP treatment. The mRNA levels of *Nrf2*, *Nqo1*, and *Gclc* were significantly reduced in BAP31-LKO hepatocytes than in WT controls 24 h after APAP administration, suggesting that reduced *Nrf2* mRNA stability may be contributed to reduced Nrf2 signaling activation in BAP31-LKO mice (Figure 6A). Accordingly, actinomycin D was added to the culture of primary hepatocytes, and mRNA decay was followed over time. The half-life of *Nrf2* mRNA is ~1.3 h in WT hepatocytes and ~40 min in BAP31-LKO hepatocytes, demonstrated that BAP31-deficiency decreased *Nrf2* mRNA half-life and reduced *Nrf2* mRNA stability (Figure 6B). Western blot analysis confirmed the transcriptional results, which displayed reduced protein levels of Nrf2, Nqo1, and Gclc in BAP31-LKO hepatocytes than in WT hepatocytes (Figure 6C). These results demonstrated that BAP31-deficiency attenuated Nrf2 signaling activation, reduced the induction of the downstream of antioxidant genes, therefore enhanced APAP-induced oxidative stress and liver injury in mice.

It was reported that Nrf2 signaling was modulated by a variety of miRNAs, which have been suggested to play a critical role in APAP-induced hepatotoxicity [17]. Thus, *miR-223* and *miR-34a* expression levels were determined. *miR-223* was reduced in BAP31-LKO mice than in WT controls with or without APAP treatment. No significant difference in *miR-34a* expression levels was determined between WT and BAP31-LKO mice (Figure 6D).

### 2.7. BAP31-Deficiency Increased Mitochondrial Dysfunction

**Figure 7:**
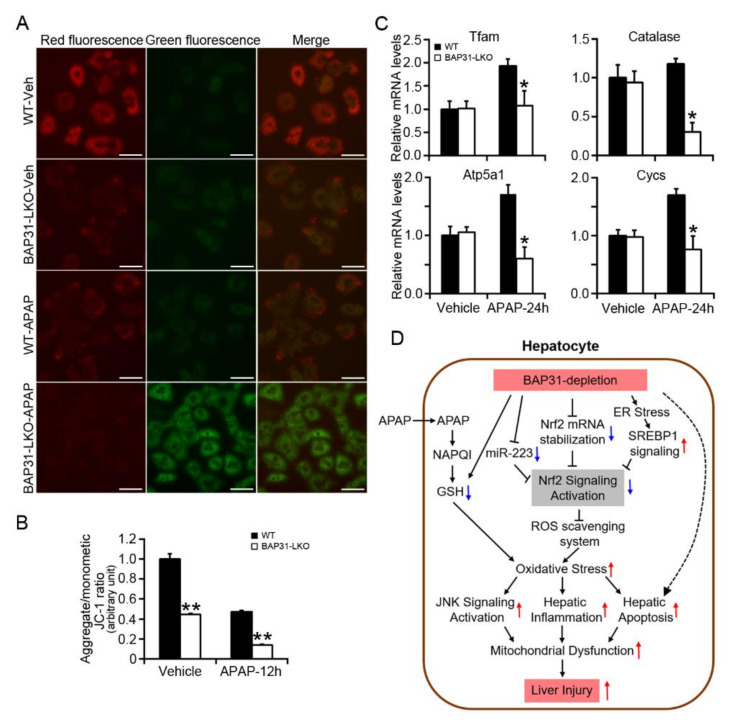
BAP31-deficiency increased mitochondrial dysfunction. (**A**) Representative images of JC-1 staining in primary hepatocytes treated with APAP (5 mM) for 24 h. Scar bar = 50 μm. (**B**) Quantitative analysis of red/green fluorescence ratio in each group. All values are shown as mean ± SE from 12 independent images captured in each group. **, *p* < 0.01, BAP31-LKO compared to WT hepatocyte. (**C**) The mRNA levels of genes related to mitochondria function were determined in WT and BAP31-LKO mice livers upon APAP administration. *n* = 5 per group. Data are expressed as mean ± SE. *, *p* < 0.05, BAP31-LKO compared to WT mice. (**D**) The working model depicting that BAP31-depletion decreased *Nrf2* mRNA stability, and Nrf2 signaling activation reduced the antioxidant response and enhanced APAP-induced oxidative stress, hepatic inflammation, and apoptosis, eventually amplified APAP-induced liver injury in mice.

In order to explore whether BAP31-deficiency affected mitochondria function, the mitochondrial membrane potential (MMP) was determined in primary hepatocytes. When MMP is high, the JC-1 fluorescent probe accumulates in the matrix of mitochondria and aggregates to form a polymeric complex that emits red fluorescence. If the MMP reduces, JC-1 cannot aggregate and exists as a JC-1 monomer, which produces green fluorescence. The change in the ratio of red to green fluorescence is used as an indicator of mitochondria condition. JC-1 fluorescent color changed from red to green along with APAP administration, which exhibiting lower red/green (aggregate/monometic) ratio, suggesting the loss of MMP and the induced mitochondria damage in primary hepatocytes (Figure 7A). Compared to WT controls, BAP31-LKO hepatocytes exhibited a reduced red/green fluorescence ratio with or without APAP administration, demonstrated that BAP31-deficiency reduced MMP in primary hepatocytes (Figure 7B). In addition, the transcriptional levels of genes related to mitochondria function were evaluated. After APAP treatment, the mRNA levels of *mitochondrial transcription factor A* (*Tfam*), *catalase*, *ATP synthase F1 subunit α* (*Atp5a1*), and *cytochrome c, somatic* (*Cycs*) were reduced in BAP31-LKO mice than in WT controls (Figure 7C), demonstrated that BAP31-deficiency-induced mitochondrial dysfunction in mice livers.

## 3. Discussion

The current study reported that BAP31-depletion caused GSH depletion, increased oxidative stress and DNA damage, promoted JNK signaling activation, hepatic inflammation, and mitochondrial dysfunction. Furthermore, loss of BAP31 decreased *Nrf2* mRNA stability and *miR-223* expression, attenuated Nrf2 signaling activation and antioxidant genes expression, which enhanced APAP-induced liver injury and hepatotoxicity. Herein, we hypothesize that BAP31-deficiency reduces *Nrf2* mRNA stabilization and *miR-223* expression, accompanied by the increased ER stress and SREBP1 signaling, which inhibits Nrf2 signaling activation and reduces the antioxidant response, and increases oxidative stress in the liver, thus leads to JNK signaling activation, hepatic inflammation, and apoptosis, results in mitochondrial dysfunction, eventually enhances APAP-induced liver injury in mice (Figure 7D).

As the most important antioxidant transcription factor, Nrf2 plays a key role in the activation of the antioxidant defense system, has been reported to be involved in the scavenging of ROS in APAP-treated mice [18]. Upon encountering oxidative stress, Nrf2 translocates from the cytosol to the nucleus, where it is recruited to the antioxidant response element (ARE) binding sites and induces a battery of antioxidant genes, including *Nqo1*, *Ho-1*, and *Gclc* [19]. Nrf2 knockout mice are more susceptible to APAP-induced liver damage due to the reduced expression of ARE-regulated drug-metabolizing enzymes and antioxidant genes [20]. In contrast, constitutive activation of Nrf2 by genetic disruption of Kelch-like ECH-associated protein 1 (Keap1) in the liver significantly reduced APAP-induced liver damage by inducing the antioxidant genes of *Nqo1*, *GST*, and *Gclc* [21]. The post-transcriptional modality to regulate *Nrf2* mRNA, which stabilized *Nrf2* mRNA, or enhanced *Nrf2* mRNA maturation and promoted the nuclear export, suggested the alternative intervention to modulate Nrf2 signaling response in disease [22]. HepG2 cells expressing Cyp2e1 showed increased *Nrf2* mRNA stability, promoted *Nrf2* mRNA and protein expression, exhibited increased Nrf2-ARE binding activity, and Nrf2-regulated genes expression of *Gclc* and *Ho-1* [23]. In line with these observations, BAP31-LKO mice showed reduced *Nrf2* mRNA stabilization and Nrf2 signaling activation, which decreased the antioxidant response upon APAP challenge, hence enhanced APAP-induced hepatotoxicity.

Mutations in BAP31 gene-altered ER morphology and caused disorganization of the Golgi apparatus, highlighting the important roles of BAP31 in maintaining ER homeostasis [24]. ER contains numerous types of chaperons, controlling newly synthesized protein folding. The properly folded protein will be transported to the Golgi apparatus and then be modified for further translocation to their destined sites [25]. The misfolded or unfolded proteins aggregate in the ER lumen, where they will be degraded via ER-associated degradation. Environmental and genetic factors that disrupt ER function cause an accumulation of misfolded and unfolded proteins in the ER lumen, which triggers the initiation of ER stress. BAP31 promoted the retrotranslocation of unfolded protein from the ER to cytosol and enhanced ER-associated degradation via 26S proteasome system, facilitated to reduce cellular unfolded protein accumulation [26]. BAP31 interacts with Tom40 protein within ER-mitochondria contact site, acts as a key factor for maintaining mitochondrial homeostasis [27]. The specific deficiency of BAP31 in hepatocytes increased unfolded protein accumulation, which induced ER stress and the related oxidative stress [9,10], consequently enhanced APAP-induced liver injury in mice livers.

A consequence of ER stress is the accumulation of ROS, which promotes oxidative stress, and induces Nrf2 signaling activation [28,29]. Enhanced ER stress initiated the autophosphorylation of protein kinase-like endoplasmic reticulum kinase (PERK). PERK phosphorylated Nrf2 and led to its nuclear localization, up-regulated Ho-1 expression, and contributed to cellular redox homeostasis [30]. Maybe this is the reason why we detected enhanced transcriptional levels of *Nrf2*, *Gclc*, and *Nqo1* in BAP31-LKO mice at normal conditions, aiming to combat the enhanced ER stress and maintaining ER homeostasis in hepatocytes. Along with APAP treatment, BAP31-deficiency kept on reducing Nrf2 signaling and overwhelmed ER stress effects of inducing Nrf2 signaling, thus exhibited reduced Nrf2 signaling after 24 h APAP treatment in mice livers. The possible molecular mechanism is that BAP31 deficiency reduced *Nrf2* mRNA stabilization, which inhibited Nrf2 signaling activation upon APAP administration.

Previously we reported that BAP31-deficiency induced SREBP1 signaling activation [9]. Chronic administration of high fructose diet activated SREBP1 signaling and inhibited Keap1/Nrf2 antioxidant signaling, suggesting the negative mutual relationship between Nrf2 and SREBP1 signaling pathways [31]. Vine tea polyphenol inhibited hepatic lipogenesis via reducing the mature levels of SREBP1C and increased the protein levels of Nrf2, Gclc and Ho-1, further suggested the negative roles of SREBP1 on Nrf2 signaling [32]. These observations hinted that enhanced SREBP1 signaling due to increased ER stress might modulate Nrf2 signaling negatively and contributed partly to APAP-induced liver injury in BAP31-LKO mice (Figure 7D). Additionally, we reported that BAP31-deficiency reduced *miR-223* expression in mice livers, which may contribute to reducing Nrf2 signaling activation. Ellagic acid elevated *miR-223* expression, down-regulated Keap1 mRNA and protein levels, and up-regulated Nrf2 signaling activation in HepG2 cells [33]. All of these effects contribute to reducing Nrf2 signaling activation in mice livers.

It is well known that aseptic inflammatory response also plays a key role in the pathogenesis of APAP-induced hepatotoxicity. The inflammatory response of the immune system determines the severity of hepatotoxicity [34]. Critical to the inflammatory response is the activation of IL-1β by the molecular complex of inflammasome [35]. Therefore, excessive pro-inflammatory cytokines, such as IL-1β, IL-6, and TNFα, are considered to be the precursors of APAP-induced liver injury, and these pro-inflammatory cytokines are considered to affect the process of APAP-induced liver injury [36]. Currently, we demonstrated that the macrophage pro-inflammatory markers of *Saa1*, *Saa2*, *Mcp1*, *Mip1α*, and *Cxcl-10* were significantly induced in BAP31-LKO mice, suggesting that BAP31-deficiency increased the inflammatory response in the liver. APAP-induced liver inflammation involves JNK activation. The activated JNK signaling pathway also acts on mitochondria, promotes the production of intracellular ROS, and exacerbates the generation of oxidative stress [37], further promotes mitochondria permeability transition, and inhibits mitochondria bioenergetics, which is in agreement with our observation (Figure 7A,C). BAP31-LKO mice exhibited enhanced p-JNK levels and inflammatory response, aggravated APAP-induced liver injury. This observation is consistent with the previous studies in neuroinflammation [38] and in HFD-induced hepatic inflammation [9].

N-acetylcysteine (NAC) helps to replenish GSH reserves by providing cysteine, which is the essential precursor of GSH production and is the only Food and Drug Administration-approved antidote for the treatment of potential hepatotoxicity due to APAP overdose [39]. Various researches suggested that antioxidants protect against APAP-induced liver disease. Rosmarinic acid possesses the biological activities of antioxidant and anti-inflammation effects, significantly decreased ALT and AST levels, and ameliorated APAP-induced liver injury via RACK1/TNFα signaling in mice [40]. Mitochondria-targeted antioxidant Mito-Tempo protected against APAP hepatotoxicity, attenuated mitochondrial oxidative stress, and mitochondrial dysfunction in a JNK-dependent manner, suggesting the promising therapeutic agent for APAP overdose patients [41]. The current study reported that BAP31 deficiency reduced antioxidant response, promoted APAP hepatotoxicity via attenuating Nrf2 signaling, suggesting the important roles of BAP31 mediating APAP hepatotoxicity. Our previous study reported that fatty liver or liver steatosis reduced BAP31 expression in the liver and promoted ER stress in HFD-induced obese mice model [9]. Nonalcoholic fatty liver disease (NAFLD) favored APAP hepatotoxicity, and the pre-existent mitochondrial dysfunction associated with NAFLD also could be involved [42]. So we suggested that genetic or pharmacological activation of BAP31 expression in NAFLD patients received APAP description could attenuate the induced liver toxicity. Regarding BAP31 function on APAP overdose treatment is interesting and should be warranted in our future studies.

In summary, our findings indicated that the liver-specific deficiency of BAP31 enhanced APAP-induced liver toxicity in mice due to the weakening of Nrf2 signaling and the increased oxidative stress, pointing to the protective roles of BAP31 in the pathogenesis of APAP-induced hepatotoxicity, suggesting BAP31 as the potential therapeutic target for liver diseases caused by APAP overdose.

## 4. Materials and Methods

### 4.1. Animals

Mice harboring a floxed allele in which loxP sites flanked exon 3 of BAP31 were crossed with the Alb-Cre expressing strain to obtain a liver-specific knockout of BAP31 (BAP31-LKO) [9]. The mice harboring a floxed allele of BAP31 were indistinguishable from pure C57BL/6 mice and were used as the control mice (WT) in the current study. Mice were kept in an environmentally controlled room with a 12 h light/dark cycle and free access to food and water. All procedures were conducted in accordance with the Guide for the Care and Use of Laboratory Animals (8th edition. Washington (DC): National Academies Press (USA); 2011) and were approved by the institutional review board of Northeastern University (NO. 2019(018)) in October 15 2019. The mouse models are kept in the university animal facility and available to the research community for independent assessment.

### 4.2. APAP Administration

Age-matched male WT and BAP31-LKO mice fasted overnight were injected with APAP intraperitoneally (300 mg/kg body weight) (J&K Scientific Ltd., Beijing, China) [43]. The control group was given the same volume of saline. Mice were sacrificed under isoflurane anesthesia 0, 6, 24, 48, and 72 h post APAP administration. Sera and liver tissues were harvested. A portion of liver tissue was fixed immediately in 4% buffered formalin for histological analysis. The remaining was flash-frozen in liquid nitrogen and stored at −80 °C for future studies.

### 4.3. Survival Rate Analysis

Age-matched male WT and BAP31-LKO mice fasted overnight were injected with a lethal dose of APAP (600 mg/kg body weight) intraperitoneally [44]. The survival rate of WT and BAP31-LKO mice was determined till 108 h post APAP administration.

### 4.4. Primary Hepatocyte Isolation and Cell Culture

Mouse primary hepatocytes were isolated from 16- to 18-week-old male WT and BAP31-LKO mice using a two-step collagenase perfusion as before [45]. A total of 1 × 10^6^ cells/well in 2 mL of complete medium (DMEM, 10% FBS, 100 nM insulin, and 1 μM dexamethasone) were seeded on collagen-coated plates and maintained in maintenance medium (DMEM, 0.1% BSA, 100 nM insulin, and 1 μM dexamethasone). Twenty-four h after plating, primary hepatocytes were treated with 5 mM APAP at the indicated time. HepG2 cells were infected with shRNA lentivirus targeting BAP31 and the scrambled non-target negative control based on the manufacturer’s instructions (Novobio Scientific, Shanghai, China). Cells were maintained in DMEM medium supplemented with 10% FBS and then treated with APAP (1 or 5 mM) for 24 h. Or treated with sulforaphane (10 μM) and APAP (5 mM) for 24 h, then total RNA was extracted, and the relative mRNA levels were evaluated.

### 4.5. Liver Enzymes, Glutathione, and Glutathione Reductase Activity Measurement

Hepatotoxicity was determined by measuring serum ALT and AST levels using the reagent kits according to the manufacturer’s instructions (Nanjing Jiancheng Biomedical Company, Nanjing, China). Frozen liver tissues were lysed, and hepatic GSH, GSSG, MDA, and SOD concentrations were quantified according to the manufacturer’s instructions (Nanjing Jiancheng Biomedical Company, Nanjing, China). Glutathione reductase activity was determined by using the assay kit from Beyotime Biotechnology (S0055, Shanghai, China).

### 4.6. Histopathology

Liver tissues fixed in 4% neutral-buffered formalin were embedded in paraffin, cut into 5 μm-thick sections, and stained with hematoxylin and eosin (H/E) according to a standard protocol [46]. H/E-stained sections were examined using a LEICA DMI3000 B inverted microscope (Leica Biosystems, Wetzlar, Germany) and used for necrosis scoring. Necrotic areas were quantified by Image J software. The percentage of liver necrosis was measured as a percentage of the total area analyzed.

### 4.7. TUNEL Assay

The paraffin-embedded liver sections were deparaffinized and subjected to heat-mediated antigen retrieval. TUNEL assay was performed using the One-Step TUNEL Apoptosis Assay Kit (C1088; Beyotime Biotechnology, Shanghai, China). DAPI was used to stain the nuclei. Images were taken with an inverted fluorescence microscope using the appropriate fluorescence filters. The number of TUNEL-positive cells in each group were counted on five 100× fields selected from each section randomly. An apoptotic index (the number of nuclei labeled by the TUNEL method/the number of total nuclei) was calculated [47].

### 4.8. Assessment of Mitochondrial Membrane Potential

Flouroprobe 5,5′,6,6′-tetrachloro-1,1′,3,3′-tetraethylbenzimidazol-carbocyanine iodide (JC-1) was employed to measure mitochondrial depolarization in primary hepatocytes accordingly [48]. Following treatment with 5 mM APAP for 24 h, cells were incubated with JC-1 (10 μg/mL) for 20 min at 37 °C, washed two times with the staining buffer, and then placed in 1 mL of culture medium for imaging. Red fluorescence (JC-1 as aggregates at high membrane potentials) and green fluorescence (JC-1 as a monomer at lower membrane potentials) were monitored under a fluorescence microscope (LEICA DMI3000 B, Leica Biosystems, Wetzlar, Germany). Mitochondrial depolarization was indicated by a decrease in the red/green fluorescence intensity ratio.

### 4.9. RNA Extraction, Reverse Transcription, and qRT-PCR

Total RNA was isolated using TRIzol^®^ reagent. RNA concentration and quality were assessed using Nanodrop Microvolume Spectrophotometers and gel electrophoresis, respectively. Two micrograms of total RNA were converted to cDNA using the GoScript^TM^ Reverse Transcription System (Promega Corporation, Madison, WI, USA). qRT-PCR was carried out using SYBR Green Master Mix (cat. #B21203. Bimake, Shanghai, China) according to the manufacturer’s instructions with a CFX96 Touch^TM^ Real-Time PCR Detection System (Bio-Rad Laboratories, Hercules, CA, USA). The cycling conditions were as follows: initial denaturation at 95 °C for 10 min, followed by 45 cycles of 95 °C for 10 s, 60 °C for 20 s, and 72 °C for 20 s. Liver miRNAs were extracted using RNAeasy^TM^ Small RNA Isolation Kit according to the manufacturer’s protocol (Beyotime Biotechnology, Shanghai, China). miRNA reverse transcription and the relative expression levels were determined using an miRNA First Strand cDNA synthesis Tailing Reaction Kit accordingly (cat. #B532451, Sangon Biotech, Shanghai, China) [49]. The unique forward primers were 5’-CGTGTATTTGACAAGCTGAGTT-3’ for miR-223-5p, and 5’-TGGCAGTGTCTTAGCTGGTTGT-3’ for miR-34a-5p. The universal reverse primer was provided by the manufacturer. Forward and reverse primers for U6 were 5’-ATTGGAACGATACAGAGAAGATT-3’ and 5’-GGAACGCTTCACGAATTTG-3’. The cycling conditions were as follows: initial denaturation at 95 °C for 30 s, followed by 40 cycles of 95 °C for 5 s, 60 °C for 30 s. U6 was selected as the internal control for miRNAs quantification. Data were analyzed using the 2^−∆∆Ct^ method. All the primer sequences are shown in Supplemental Table S1.

### 4.10. RNA Stability Assay

The RNA stability was determined according to the previous publication [50]. Briefly, mouse primary hepatocytes were isolated and plated 16 h before the treatment of actinomycin D (10 μg/mL) (Selleck Chemicals, Houston, TX, USA). The cells were collected after treatment at 0, 1, 2, 3, 4, and 6 h. Total RNAs were extracted. qRT-PCR was performed to assess gene expression. The mRNA levels of *18S* rRNA were used as the internal control. The percentage of the remaining mRNA was normalized at h 0 for both groups. The half-life of *Nrf2* mRNA was calculated by using a one-phase exponential decay curve analysis GraphPad Prism v8.3.0 (GraphPad Software, San Diego, CA, USA).

### 4.11. Western Blot Analysis

An equivalent amount of protein extracts from liver tissues, primary hepatocytes, or HepG2 cells were resolved by SDS-PAGE and then transferred to the PVDF membrane. The membrane was blocked with 5% non-fat dry milk or 5% BSA in TBST. Membranes were incubated with primary antibodies at 4 °C overnight, following by incubation with HRP-conjugated secondary antibody for 1 h at room temperature. The results were visualized with Bio-Rad ChemiDoc^TM^ imaging systems using an ECL detection kit (Bio-Rad Laboratories, Hercules, CA, USA). The antibodies source and dilution are shown in Supplemental Table S2.

### 4.12. Statistical Analysis

Quantitative data were presented as mean ± SE. The survival rate analysis was assessed with a log-rank test (GraphPad Prism v8.3.0, GraphPad Software, San Diego, CA, USA). Other statistic differences were determined using a one-way ANOVA followed by a Duncan’s Multiple Range post hoc test. All statistical tests with *p* < 0.05 were considered statistically significant.

## Data Availability

All data are presented and available in the manuscript. The corresponding author is available to provide any additional information on data presented in this study.

## Supplementary Materials

The following are available online at https://www.mdpi.com/article/10.3390/ijms221910788/s1.
