# Supplementary material for: Hepatocyte-Specific Deficiency of BAP31 Amplified Acetaminophen-Induced Hepatotoxicity via Attenuating Nrf2 Signaling Activation in Mice
Source: Int J Mol Sci. 2021 Oct 5;22(19):10788. doi: 10.3390/ijms221910788 (PMC8509202; doi:10.3390/ijms221910788)
Supplement: Supplementary file 1 [file ijms-22-10788-s001.zip › ijms-1332707-supplementary.pdf]

Table S1. Primer sequences for real-time PCR

Genes	Forward (5'→3')	Reverse (5'→3')
Atp5a1	GAGAGCAGCCAAGATGAACG	GACACGGGACACAGACAAAC
Catalase	AGGAGGCAGAAACTTTCCCA	GGCCCTGAAGCATTTTGTCA
Cxcl-10	CCAAGTGCTGCCGTCATTTTC	GGCTCGCAGGGATGATTTCAA
Cyp2e1	CGTTGCCTTGCTTGTCTGGA	AAGAAAGGAATTGGGAAAGGTCC
Cyp4a10	AGAACTTCCCAAGTGCCTTTC	GCAAACCATACCCATTAGCCTTT
Cycs	GTTCAGAAGTGTGCCCAGTG	GTCTGCCCTTTCTCCCTTCT
Gclc	GGGGTGACGAGGTGGAGTA	GTTGGGGTTTGTCCTCTCCC
Ho-1	AAGCCGAGAATGCTGAGTTCA	GCCGTGTAGATATGGTACAAGGA
IL-6	AGTTGCCTTCTTGGGACTGA	CAGAATTGCCATTGCACAAC
IL-1β	GACCTTCCAGGATGAGGACA	AGCTCATATGGGTCCGACAG
Gpx1	GTGCAATCAGTTCGGACACCA	CACCAGGTCGGACGTACTTG
Gpx2	GCCTCAAGTATGTCCGACCTG	GGAGAACGGGTCATCATAAGGG
Mcp1	TTAAAAACCTGGATCGGAACCAA	GCATTAGCTTCAGATTTACGGGT
Mip1α	TTCTCTGTACCATGACACTCTGC	CGTGGAATCTTCCGGCTGTAG
Nox2	TGTGGTTGGGGCTGAATGTC	CTGAGAAAGGAGAGCAGATTTCG
Nqo1	AGGATGGGAGGTACTCGAATC	AGGCGTCCTTCCTTATATGCTA
Nrf2	TCTTGGAGTAAGTCGAGAAGTGT	GTTGAAACTGAGCGAAAAAGGC
Saa1	TCATTTGTTCACGAGGCTTTC	CTTTGAGCAGCATCATAGTTCC
Saa2	TGGCTGGAAAGATGGAGACAA	AAAGCTCTCTCTTGCATCACTG
Sod1	AACCAGTTGTGTTGTCAGGAC	CCACCATGTTTCTTAGAGTGAGG
Sod2	CAGACCTGCCTTACGACTATGG	CTCGGTGGCGTTGAGATTGTT
Tfam	GCAGCTAACTCCAAGTCAGC	CCGAATCATCCTTTGCCTCC
TNFα	CCCTCACACTCAGATCATCTTCT	GCTACGACGTGGGCTACAG
Trx-1	CATGCCGACCTTCCAGTTTTA	TTTCCTTGTTAGCACCGGAGA
18S	AGTCCCTGCCCTTTGTACACA	CGATCCGAGGGCCTCACTA

Table S2: List of antibodies used in this study

Antibody	Isotype	Cat#	Source	Dilution
Nrf2	Rabbit IgG	#12721	CST	1:1000
Nqo1	Goat IgG	ab2346	Abcam	1:2000
Gclc	Rabbit IgG	ab53179	Abcam	1:2000
TNFα	Rabbit IgG	#3707	CST	1:1000
GSTA1	Rabbit IgG	abs136034	Absin	1:2000
BAP31	Goat IgG	ab10924	Abcam	1:2000
JNK	Rabbit IgG	#9252	CST	1:1000
p-JNK	Rabbit IgG	#4668	CST	1:1000
GAPDH	Rabbit IgG	#2118	CST	1:5000

CST: cell signaling technology. Absin: Absin Bioscience Inc.
